# Conservative Medicine

**Published:** 1862-09

**Authors:** Austin Flint

**Affiliations:** Prof. of the Principles and Practice of Medicine in the Bellevue Hospital Medical College, N. Y., and in the Long Island Hospital


### CONSERVATIVE MEDICINE.

By AUSTIN FLINT, M. D.,

*Prof. of the Principles and Practice of Medicine in the Bellevue Hospital Medical College, N. Y., and in the Long Island Hospital.*

"What does the writer mean by *Conservative Medicine?*" This will be the mental inquiry of the reader when the caption of this article meets his eye. It is desirable, first of all, for the writer to explain the subject which he ventures to hope will appear to possess interest enough to lead to a perusal of the pages which are to follow.

The meaning of *Conservative Surgery* is well understood. This phrase has been sufficiently common of late years. The conservative surgeon aims to preserve the integrity of the body. He spares diseased or wounded members whenever there are good grounds for believing that by skillful management they may be saved. He resorts to mutilations only when they are clearly necessary. He weighs carefully the dangers of operations, so as not to incur too much risk of shortening life by resorting to the scalpel. By conservative medicine, I mean an analogous line of conduct in the management of maladies which are not surgical. The conservative physician shrinks from employing potential remedies whenever there are good grounds for believing that diseases will pursue a favorable course without active interference. He resorts to the therapeutical measures which must be hurtful if not useful, only when they are clearly indicated. He appreciates injurious medication, and hence does not run a risk of shortening life by adding dangers of treatment to those of disease. Such, in brief, is an explanation of the subject of this article. For the phrase *conservative medicine* I am indebted to a distinguished friend and colleague, well known as eminently a conservative surgeon.*

During the last quarter of a century a change has taken place in medical sentiment as regards surgical operations. New and grand achievements in surgery seemed formerly to be the leading objects of personal ambition. To borrow a fashionable expression, they were decidedly the rage. Boldness in the use of the knife was the trait in the character of the surgeon which was most highly admired. The history of surgery during the first third of the present century is characterized by the introduction and frequent performance of numerous formidable operations. It was customary to speak of them as brilliant, and the daring surgeon enjoyed somewhat of the *eclat* which belongs to the hero of the battle-field. This analogy was implied when one of the greatest of our American surgeons, wishing to distinguish his most brilliant exploit, styled it his Waterloo operation. The change that has taken place is marked. We hear now comparatively little of terrible operations, and of that sort of heroism which is associated with bloody deeds. What would once have been considered as a degree of courage to be admired, is now stigmatized as rashness. It is an equivocal compliment to say of a practitioner that he is a bold surgeon. The change, it may be said, is in a measure due to the fact that the great number of new operations which have been introduced since the beginning of the present century leaves but a limited range for further explorations in that direction; but this explanation will go only a little way. The change is one of sentiment. The desire is to preserve the integrity of the body, to avoid mutilations, to incur the dangers of capital operations only when they are imperatively called for—in a word, conservatism

Prof. Hamilton.*

has become the ruling principle in surgery. The most important of the most recent improvements in surgery exemplify the influence of this principle on the medical mind.

An analogous change, within the same period, has taken place in medical practice. Formerly, boldness was a distinction coveted by the medical, as well as by the surgical practitioner. "Heroic practice" was a favorite expression, consisting in the employment of powerful remedies, or in pushing them to an enormous extent. The physician emulated the surgeon in daring. The change is not less marked in medicine than in surgery. We hear now oftener of diseases managed with little or no medication, than of cases illustrating the abuse of remedies. In the treatment of many affections it is considered necessary to employ measures which, but a few years ago, it would have been considered culpable to withhold. The change, too, is here one of sentiment. We desire to preserve the vital forces, to avoid the perturbations and damaging effects of potential therapeutic agencies—in short, conservatism has become a leading principle in medicine as well as in surgery. The improved method of managing a host of affections will be found to illustrate this fact.

Before proceeding further, let us inquire how the contrast between medical practice at the present moment and a quarter of a century ago, should affect our estimation of medicine. Is medicine disparaged by the changes which have actually taken place? It is not enough to answer this question in the negative. Mutations, when they denote progress, are, of course, desirable. In so far as the contrast shows improvement, medicine at the present moment is deserving of esteem, the more, as the changes are great. It redounds to the glory of medicine that it admits of illimitable progressive changes. In this fact lies the distinctive feature of legitimate medicine as contrasted with illegitimate systems of practice. But, some one may say, is there to be no stability in medicine, no traditional authority, and is reverence for the past to have no influence? If not, where is our ground of confidence in the practice of the present day? And is it not probable that at the end of another quarter of a century mutations will have occurred twice as great as those which have taken place during the last twenty-five years? These questions are to be met fairly and squarely; let us endeavor so to meet them.

Waiving the consideration of what constitutes perfectibility in the *ars medendi*, and whether it be obtainable or not, no one will assert that medicine is now, or ever has been, in a condition not to admit of indefinite improvement. Improvement in its practical applications and results is the great end of the labors devoted, now and hitherto, to the different departments of medical knowledge, viz: anatomy, physiology, animal chemistry, materia medica, pathology, and clinical medicine. We may assume that these labors, thus far, have not been profitless, and, accordingly, that practical medicine has improved. We may assume, also, that there is abundant encouragement to continue these labors, and, hence, that further improvement is to be expected—to what extent it is vain to speculate. It necessarily follows that stability in medicine is not to be counted upon; that the doctrines of to-day have no intrinsic claim to perpetuity; that because they are now in vogue is not a sufficient reason why they should not hereafter be modified or rejected, and that there is, to say the least, no ground to deny the possibility of the changes which are to take place hereafter being a whit less than those which have already taken place. What then? There are skeptics and scoffers with regard to medicine, and there are many persons who live and thrive by promoting popular distrust of it. It may seem to be giving aid and comfort to the enemies of medicine to concede that its past history abounds in errors, that present errors doubtless abound, leaving ample scope for future improvement. Be it so. We have nothing to do with skeptics, scoffers, and charlatans. We are not called upon to repel attacks prompted by ignorance, selfishness, and deceit. Yet it is desirable, with regard not only to the interests of the medical profession, but to the welfare of humanity, that medicine should hold its proper place in popular estimation. What, then, is the attitude to be taken as regards the just claims of medicine to public consideration and confidence? A body of men in every generation, from the time of Hippocrates to the present day, in all civilized conntries, have conscientiously and industriously labored to acquire knowledge of diseases with reference to the relief of suffering and the prolongation of life. Under a host of difficulties and obstructions, many inherent in the pursuits themselves, and others proceeding from various intrinsic sources, the labors of physicians and their collaborators have continued and still continue. Now, granting that they have advanced slowly and often been led astray, where else can society ever seek for aid in the necessities of illness with a better prospect of success? Granting that they have failed, and still fail, in conferring all the benefit that it is to be desired, and that, with the purest intentions, their efforts have been sometimes not only without avail, but hurtful, should the preponderating good be therefore overlooked, and is there any rational alternative but to accept good and submit to the limitations and errors incident to existing knowledge? All that society can claim of medicine in any generation is, the capabilities of the medical science in that generation. All that society can claim of physicians is, that these capabilities shall be understood and judiciously applied. But we are opening up trains of thought which will lead us a long way from our subject, and we must abruptly return to the consideration of conservative medicine.

It is an interesting point of inquiry, whence came the influences leading to conservatism as a principle of medical practice? The answer to this inquiry would not be the same in all countries and sections. It must be admitted that in our country the earliest and fullest development of the principle was in New England. Our New England brethren are fond of dating a new order of medical ideas from the publication of an address, more than twenty years ago, by Jacob Bigelow, on the self-limited character of certain diseases. Not underrating the importance of that publication, the spirit of the oral teachings of James Jackson and John Ware has exerted on the medical mind of New England an influence which can only be appreciated by those who have experienced it. To those who have known experimently the value of their teachings, it is a source of deep regret that the influence of these admirable professors has not been more widely diffused by means of larger contributions to medical literature. British conservatists attribute much to the writings of Dr. Forbes. Among the non-medical observers of the change in practice which has taken place, some have been pursuaded that it is due to the disciples of Hahnemann, an idea too preposterous to need refutation. The truth is, we are not to look for the causes of the change exclusively in the views emanating from particular persons. It is rather a legitimate result of scientific researches in different directions. If we were to specify circumstances which have more especially been instrumental in leading to the principle of conservatism, we would mention, *first*, the abandonment of the attempt to found a system or theory of medicine after the decline and fall of Brunonianism and Broussaisism; and *second*, the study of diseases after the numerical method with reference to their natural history and laws.

Strange as it appears, the importance of determining by clinical observation the intrinsic tendencies of different diseases as the basis of therapeutics, seems to have been heretofore overlooked. Physicians have acted on the presumption that most diseases do not pursue a favorable course without treatment more or less efficient. This has been, to a still greater extent, the popular belief. The apparent proof of the success of the Hahnemannic treatment rests on this belief. What are the facts already ascertained with respect to the intrinsic tendencies of different diseases? We know that diseases in the management of which, but a few years ago, the physician would not dare to omit potent therapeutical measures, almost invariably end in recovery without any active treatment. Take, as examples, pneumonia limited to a single lobe, and acute pleurisy. It is sufficiently settled that these diseases involve very little danger in themselves, proving fatal only in consequence of complications. The practitioner, therefore, no longer feels obliged to employ blood-letting, mercurialization, cathartics, blisters, etc., in these diseases, with reference to the saving of life. The only question is, do patients pass through these diseases as well without as with such measures of treatment? Clinical observation, following up this inquiry, arrives at results which exemplify conservative medicine.

Our acquaintance with the natural history of the great majority of diseases is, as yet, very incomplete. Knowledge of the tendencies of diseases allowed to pursue their course without active treatment, is not readily acquired. We cannot conscientiously withhold remedies which we have reason to believe may prove useful. Cases are therefore to be slowly accumulated in which, from circumstances not under our control, diseases have been uninfluenced by therapeutic interference. This knowledge, it is evident, is the true point of departure for the study of the effects of remedies as regards the termination and duration of diseases. The information already obtained has rendered the use of powerful therapeutic agencies far less common than they were but a few years since. It remains to be seen hereafter what will be the further effect on medical practice of continued researches in this direction.

Conservative medicine assumes the remedial measures, according to their potency, must either do harm or good; that they can never be neither hurtful nor useful. Prior to the advent of conservatism, this important fact was not duly appreciated. Blows were leveled at diseases, but the patient was not enough considered. It did not enter sufficiently into the calculations of practitioners, that if successive blows dealt at a disease were misdirected, the effect was not lost, but injury was inflicted in proportion to their force. Hence, it must needs follow that the sick man sometimes encountered, in addition to his malady, assaults not less real because well meant. In this respect, certainly, we have evidence of progress. We are satisfied that we do not err in saying that the most judicious practitioners of the present day accept the following maxims of that eminently conservative physician, Chomel: *first*, that we are not so much to treat diseases, as patients affected with disease; and *second*, that not to do harm, is no less an object of treatment than to do good.

In defining conservative medicine, we have seen that it expresses a characteristic of the improvements in medical practice during the last twenty-five years. Let us now direct our attention to illustrations afforded by some of the different classes of remedial measures. And, first of all, blood-letting suggests itself. How great the change as regards this remedy! Twenty-five years ago it was employed as if it were an innocuous remedy. Practitioners thought much more of the risk of not resorting to it when it was needed, than of the evil of its being needlessly resorted to. Hence, they often acted on the rule inculcated by a medical writer, viz., when in doubt, use the lancet. How different the rule of treatment now! Few practitioners of the present day would resort to this remedy in any case in which its appropriateness seemed to them questionable. Why not? Because it has been ascertained to be a spoliative remedy. It causes a disproportionate loss of the corpuscular elements of the blood, which are slowly regenerated. These corpuscular elements are already deficient in many diseases. In short, anæmia and its pathological relations were very imperfectly understood a quarter of a century ago. It is clear now to every one that, if not indicated, blood-letting should never be employed. This simple statement explains, in a great measure, the comparative disuse of blood-letting. The great question now is, whether it is a remedy called for more or less frequently in the management of certain diseases, chiefly the acute inflammations. I do not propose to enter here into a discussion of this question. This much may be said: Clinical observation, which is alone competent to settle the question, has shown that it is a remedy not called for so often or to so great an extent in acute inflammations as was supposed but a few years ago. A single incidental remark with respect to blood-letting, and it is one which will to other remedies: In determining its influence for good or evil by means of clinical observation, it is not enough to take into account the ratio of recoveries, and the duration of cases of disease. Blood-letting may not increase the mortality from a disease, nor protract its continuance, and, yet, prove injurious. The injury may be manifest only in the slowness of convalescence and the impaired condition of the system after recovery.

Cathartics were prescribed a quarter of a century ago much more generally and to a much greater extent than at the present time. In fact, purgation was considered as rarely out of place, whatever might be the nature or seat of the disease. This harmonized with the notion that very many diseases originated in, and nearly all were liable to be perpetuated by, causes acting within the alimentary canal. Abernethy's views of the constitutional origin of local diseases were generally received and acted upon, and with him the constitution and the bowels were almost convertible terms; constitutional treatment consisting in the nightly blue pill and the morning black draught. The great Sir Astley Cooper quoted with approbation the quaint saying of an old Scotch doctor, who declared that fear of God and keeping the bowels open were the chief requisites of duty for safety in this world and the world to come! The importance of purgation became deeply rooted in popular sentiment. Cathartic pills or potions were considered indispensable in every household, and it would hardly express the frequency with which they were used, to say that family devotions were far less common. These were the days when, as Stokes remarks, more truly than chastely, doctors seemed to have always in their minds "a cathartic and a pot-full of fæces." In this day, when a change has taken place as respects the employment of purgatives, physicians suffer from the fact that it takes a long time to eradicate a firmly-fixed popular notion. Not only do we find it often embarrassing to reconcile patients to a different practice, but we are expected to inquire into, and carefully examine daily, by sight and smell, the excretions of patients, when we might otherwise consult our comfort (to say nothing of dignity) by dispensing with this exercise of the senses. The objects for cathartics, as now considered, are comparatively few, consisting chiefly in the removal of constipation, and their hydragogue operation in dropsy. They are no longer given as a matter of course, without definite indications. As perturbatory and debilitating agents, they cannot but do harm if not required, and their frequent repetition conflicts with nutrition, and thereby with sustaining measures of treatment. The change, as respects this class of remedies, thus illustrates the principle of conservatism.

It is needless to remind the reader familiar with the practice current twenty-five years ago, of the frequency with which emetics were employed. Of morbid causes referred to the alimentary canal, a large share were supposed to exist in the *primæ viæ*—an expression then often used by writers and in common parlance. The same notion taken up by the public was conveyed by the homely expression "foulness of the stomach." Emetics were prescribed by physicians to remove saburral matters, and vomiting desired by patients as a cleansing operation. Severe and prolonged vomiting by lobelia, in conjunction with the vapor bath, constituted the Thomsonian practice, which in certain parts of our country, for several years, was considerably patronized. At the present time, emesis, irrespective of cases of poisoning and over-repletion, is rarely produced, excepting as incidental to the rule of remedies not prescribed for that purpose, such as the nauseant sedatives, colchicum, veratrum viride, etc. What would be thought of a practitioner now who treated cases of phthisis with emetics repeated almost daily! Yet, within the memories of physicians of twenty-five years' standing, this practice has been advocated, and, to some extent, adopted. The progress of medical conservatism has led to the abandonment of emetics as perturbatory and debilitating agents, excepting in the rare instances in which they subserve an explicit purpose.

The practice of the present time presents a striking contrast with that twenty-five years ago, as regards the use of counter-irritant applications. The physician whose professional career has already extended over that period, is sometimes reminded of the severe measures then in vogue, by the exhibition of indelible scars on the bodies of his old patients. He is not likely now to contemplate these traces of his former vigorous practice with lively gratification. Blisters, sometimes applied successively over the same space, and not diminutive in size, tartar-emetic ointment and plasters, issues, the moxa, etc., were considered as among the most efficient of the means of influencing the cure of a host of local affections. How much less frequently are they now used, and, when counter-irritation is deemed advisable, how much milder are the applications chosen! Physicians were strongly impressed with the belief that local affections were often removed by revulsion. They accepted the doctrine of Hunter, that two diseases rarely concur, and, hence, that an artificial disease is likely to effect a cure by a process of displacemont. Not only has this doctrine been disproved by pathological researches, but these have shown a large number of the local diseases formerly regarded as primary, to be the secondary or tertiary effects of morbid conditions then unknown. Bright's disease had not been dis covered, and its multitudinous pathological consequences were, of course, unintelligible. In those days solidism prevailed, and hæmatology has been since created. Physicians made no account of blood-poisons, and the old humoral notions of coction and fermentation had not been revived under the modern but equally indefinite garb of catalysis. Mr. Farr had not invented the name Zymosis, a name expressive of our ignorance, rather than conveying any precise knowledge, but, nevertheless, significant of a wide and most important leap from the doctrine of solidism; or, in other words, of a passage backward, guided by the light of modern science, to humoralism, which, as Rokitansky remarks, is simply a requisition of common sense. This change in pathological views, in conjunction with clinical observation, has led physicians to distrust, more and more, the value of counter-irritant applications, and, at all events, to conclude that severe revulsive measures are rarely called for; hence, the change in practice is in conformity to the principle of conservatism.

The contrast as regards the use of mercury affords a signal instance of progressive change. The remarkable efficacy of this remedy in certain affections naturally led to the expectation of its utility in many diseases. Mercurialization being a disease, it accorded with the current belief of the incompatibility of different affections, to suppose that it displaced other diseases. It was considered as *par excellence* an *alterative* remedy; and what a latitude for imagined results was afforded by that title! Moreover, its supposed special action on the liver accorded with the notion that the secretion of bile had much to do with morbid phenomena. The relief or prevention of portal congestion was incidental to its hepatic effects. It lessened exudations; it promoted the absorption of morbid products; it altered the secretions; it dispelled local engorgements, and, by exciting stomatitis, it acted by way of revulsion. Waiving here, as in the other instances, discussion of the actual value of this remedy, the extravagance of the views formerly entertained is now sufficiently evident. The statements of those who have made war upon this article of the materia medica, and the popular prejudices thereby produced, are equally, or still more, extravagant; but it is a remedy potent for harm when inappropriate, as it is powerful for good when indicated; and, therefore, the great change that has taken place as regards its use exemplifies conservatism.

These examples are sufficient to show how conservative medicine is illustrated by recent improvements as regards the employment of particular therapeutic measures. They furnish evidence of immense progress in practial medicine. Let not this statement be misunderstood. The improvements which have been noticed consist in the restricted use of bloodletting, cathartics, emetics, counter-irritants, and mercurials. Does the restricted use of these measures detract from their real therapeutic value? Not at all. Medicine has, by no means, repudiated them. She employs them with better judgment and discrimination; thus, availing herself of the good they can accomplish, she escapes the evils arising from their injudicious and indiscriminate use.

If we look at the progress of medicine during the last quarter of a century from another point of view, we find additional examples of conservatism. Regarding it exclusively from the point of view already taken, it appears that, in proportion as the practice of medicine has improved, reliance on certain active or heroic measures of treatment has diminished. This is true, but it is not the whole truth. Some measures are employed with much more freedom now than a few years ago. The use of opium and alcoholic stimulants, in certain diseases, affords the most striking illustrations of this truth. These instances also exemplify the principle of conservatism. Opium and alcohol, in excessive doses, occasion immediate disorder, of more or less gravity, and may destroy life. But given so as not to incur any risk of these effects, they do not conflict with conservatism, because their operation is transient, and, unless their use be continued, they do not leave behind them damaging effects. Given in quantities which are comfortably borne, they certainly do not impair the vital forces by perturbation, by loss of fluids, by affecting the constitution of the blood, or by inducing local changes, as do the measures previously noticed. This statement, of course, has nothing to do with the ulterior consequences, moral and physical, of intemperance or opium-eating. Here, too, as in other instances, discussion of the *modus operandi* of remedies is waived. Most physicians will agree in the statement that, when indicated as remedies, opium and alcohol sustain the vital forces. In this respect they are positively conservative. But a point of distinction is, when not indicated, if given within certain limits, and not continued, they are neither spoliative, exhausting, disturbing, nor disorganizing, as are various other measures, and, therefore, not, like the latter, even then, antagonistical to conservative medicine.

The contrast between the practice of medicine now and twenty-five years ago is not less marked, as regards the use of opium and alcohol, than as regards the restricted employment of other measures. Let the practitioner, who has seen service for a quarter of a century, consider what a responsibility he would once have taken in treating cases of pneumonia with brandy and opium, to say nothing of the continued fevers. The wonderful tolerance of these remedies in certain cases of disease is a recent discovery. Let the same practitioner consider whether he would once have ventured on a hundred grains or more of opium *per diem* in a case of peritonitis, or grain doses of the sulphate of morphia hourly, continued for several days, in a case of dysentery. Let him consider whether, at the commencement of his career, with the fulminations of Broussais on incendiary practice resounding in his ears, he ever dreamed of the propriety of giving quarts of spirits daily to fever patients, and of finding the frequency of the pulse diminished, and the mind become more clear under this heavy stimulation!

If we turn from remedial measures to dietetics, we find that the improvement which has taken place in practice contributes to the illustration of conservative medicine. In fact, conservatism is, perhaps, not less conspicuous in the contrast as respects the diet of the sick than in any other point of view. In cases of fever, and all acute diseases, twenty-five years ago, it was generally deemed an essential part of the treatment to withhold alimentary supplies. It was a frequent saying to patients who craved food, that to allow it would be to nourish the disease. In chronic affections, too, the diet was usually much restricted. It was believed that a large majority of diseases were attributable, directly, to dietetic imprudences, and that the over-ingestion of food, during the progress of diseases, was, of all indiscretions, the most prolific of evil. Physicians seemed to loose sight of the plain fact that the vital powers must languish in proportion as the alimentary supplies fall below the wants of the system, and that death may be produced by starvation in disease as well as in health. At the present time, a nutritious diet is considered as highly important in the management of fevers, as well as in diseases which tend to destroy life by exhaustion, and most physicians appreciate the importance of keeping the body well nourished in chronic affections.

Incidentally a point for remark is here suggested. Twenty-five years ago disorders of digestion, grouped under the name dyspepsia, were extremely frequent. Dyspepsia was the popular malady of the day. The number of dyspeptics, of late years, has greatly diminished. The malady is comparatively infrequent. Why is this? I believe it to be explained, in a great measure, by the fact that in the matter of eating, instinct has regained its rightful supremacy. We do not hear so much now, as then, of the liabilities to dietetic errors. Physicians are not so ready to attribute diseases to some imprudence at the table. The subject is not brought to the minds of the people by means of conversation, popular books on diet, public lectures and sermons. The healthy man no longer sits down to dinner with fear and trembling lest he should eat too much, or indulge in improper articles of food. There are fewer patients who hold to the fanatical notion, that moral and physical health requires the demand of the system for food in sufficient quantity and variety, as expressed by hunger and appetite, to be resisted; and that the welfare of body and mind is promoted by living on a poor and insufficient diet. We rarely, now-a-days, hear the injunction, which was once impressed upon all who would preserve health, to adopt the habit of always rising from the table hungry. Nature and common sense have triumphed over these absurd ideas, and, among other advantages, dyspeptic ailments, which formerly tormented so many persons, have wonderfully diminished.

Recurring to the definition of conservatism in medicine, it suffices to say that it means the preservation of the vital forces. It is a principle in medical practice, covering everything which prevents impairment of, or tends to develop and sustain, the powers of life. The terms "vital forces" and "powers of life," although they are not readily explained, have a practical meaning, which is well enough understood, and it is unnecessary to enter into an explanation of them. It has been the object, in the foregoing pages, to give an exposition of conservative medicine, and to show that conservatism, in the sense in which the term is now used, is a distinguishing feature of medical practice at the present time, as contrasted with the practice which prevailed twenty-five years ago. The development and adoption of this principle have been seen to be results of progress of medical knowledge, and the circumstances which seem especially to mark the beginning of the changes illustrating the principle are, abandonment of attemps to reduce the practice of medicine to a system, after the failure of the latest, viz: Broussaisism, and the study of the natural history of diseases, as inaugurated by Louis. It is by no means, however, intended to ignore the fact that the cultivation of all the branches of medical knowledge has powerfully co-operated to the same end. The changes which have taken place during the last quarter of a century have not been due to a prior recognition of the principle of conservatism; but now that the changes have occurred, we find conservatism to be common alike to all, binding them together, and constituting their most striking characteristic. Having reached the principle thus analytically, are we not bound to recognize it as a fixed principle of medical practice, and one possessing great practical importance? Assuming it to be such, the remainder of this article will be devoted to its applications in the management of different forms of disease. And, first, let us consider the application of conservatism to the treatment of patients with inflammatory.

Theoretical views led to the measures called *antiphlogistic* in cases of inflammation. These measures, consisting of general and local blood-letting, cathartics, and rigid or restricted diet, were considered as antagonizing the state of inflammation; not unfrequently arresting its progress, and, when not successful in this end, diminising its severity, limiting its morbid effects, and abridging its duration. As already remarked, the injury which these measures are capable of doing was overlooked; and, on the other hand, all will admit that their efficacy, in effecting the objects just stated, was greatly overestimated. Clinical experince has shown that we cannot rely upon these measures to arrest the progress of inflammation. Admitting the possibility or probability of success in a small proportion of cases, we are not justified in exposing patients to the injury produced if these measures do not succeed, when the chances are few that they will prove successful. This statement expresses a rule of conservatism applicable to all potent measures employed in any disease as abortive measures of treatment. Measures not impairing the vital forces are allowable, even when the probability of success is small. Opium, for example, is admissible as an abortive remedy when blood-letting is clearly inadmissible. But measures which, if not successful, will do harm, are only to be resorted to when the chances of success preponderate over those of failure. Conservatism, therefore, does not justify the employment of the antiphlogistic treatment with a view to the arrest of inflammation, without taking the ground that they invariably fail.

(*To be continued in our next number.*)

